# Aplasia of the Maxillary Sinus, a Large Periapical Cyst in the Maxillary Arch, and a Swimming ‎and Kissing Molar: A Rare Case Report

**DOI:** 10.7759/cureus.6859

**Published:** 2020-02-03

**Authors:** Ayoob M Alnafisah, Shaul Hameed

**Affiliations:** 1 Dentistry, Qassim University, Qassim, SAU; 2 Oral Surgery and Maxillofacial Radiology, Qassim University, Qassim, SAU

**Keywords:** aplasia, cone beam computed tomography, hypoplasia, maxillary sinus

## Abstract

Aplasia of the maxillary sinus and hypoplasia are rare conditions that can trigger symptoms such as headaches and altered speech. Most patients are asymptomatic, but given the role of these symptoms in the differential diagnoses for neoplasms and infection, such symptoms warrant investigating. We present a rare case of a young man with hypoplasia of the maxillary sinus on the left side with a large periapical cyst on the right side, diagnosed via a routine cone-beam computed tomography (CBCT). This case discusses the role of CBCT in investigating aplasia of the maxillary sinus.

## Introduction

Maxillary sinus hypoplasia (MSH) is an uncommon dental condition. Computed tomography (CT) scans provide valuable data about the anatomic details of the paranasal sinuses. MSH may be misdiagnosed as an infection or a neoplasm of the maxillary sinuses. The maxillary sinuses start their growth in the third month of intrauterine life [1-2]. At birth, the volume of the sinus is 6x8 mm. The size increases by 2 mm in the lateral and vertical sizes and by 3 mm in the anteroposterior dimension in the eighth year. At 10 years of age, the lower limit of the maxillary sinus and the nasal cavity floor are on the same level and growth lasts until puberty [3].

Paranasal sinus hypoplasia is also a rare condition [4]. A review of the literature revealed that only six cases of hypoplasia have been reported to date [5]. Most frequently it is found in the sphenoid and frontal sinuses [6]. The prevalence of hypoplasia of the maxillary sinus ranges from 1.5% to 10%, but some trials have reported a prevalence of < 1.5% [6]. Most maxillary sinus aplasia patients are asymptomatic and unaware of their circumstances, and their condition is detected via conventional X-ray [4]. Some people may report concerns about facial pain, chronic headaches, and speech problems [4]. Hypoplasia of the maxillary sinus can be misdiagnosed, particularly in standard X-rays, as it can appear as mucosal thickening from infectious diseases or sinus neoplasms [4]. There is also the possibility to misdiagnose sinus atelectasis owing to chronic sinusitis as sinus hypoplasia or aplasia [7]. Bolger's classification of maxillary sinus hypoplasia classifies the condition into three types [6]. Type I is mild sinus hypoplasia with a normally developed uncinate process and well-developed infundibulum with varying degrees of sinus mucosal thickening. Type II is significant maxillary sinus hypoplasia with a hypoplastic/absent uncinate process, poorly defined or absent infundibulum, and total opacification of the affected sinus. Type III is the profound hypoplasia of maxillary sinus with an absent uncinate process.

CT scanning, cone-beam computed tomography (CBCT), and sinus endoscopy are the best diagnostic tools to reveal the underlying abnormality [8-9]. As this case illustrates, an awareness of hypoplasia of the maxillary sinus is important because of the implications involved in performing surgery in these patients. Routine X-rays can lead to an erroneous diagnosis of sinus infection because of the opacity seen in the poorly developed sinus area.

## Case presentation

A 24-year-old male patient was referred to the dental clinics of Oral and Maxillofacial Radiology at Qassim University for CBCT preparation. On physical examination, the swelling was found in the upper right tooth region. Patient history revealed resonance in the voice and an occasional headache.

**Figure 1:**
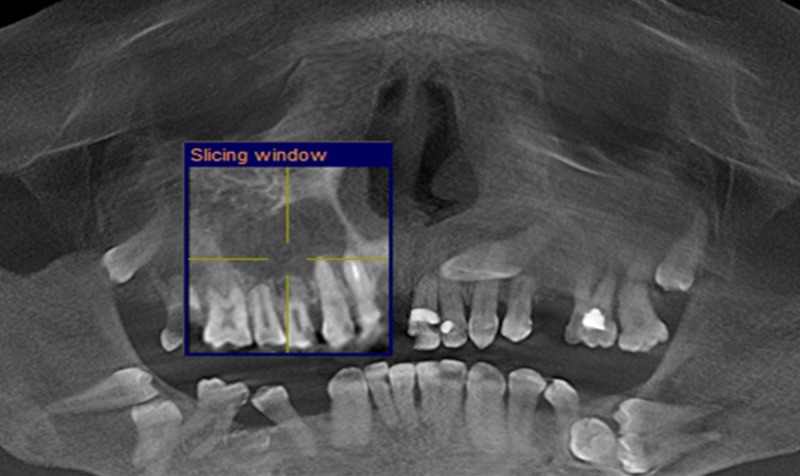
Unicystic radiolucency extending from the periapical area of 13 to the periapex of the 16 and the ‎complete absence of the maxillary sinus on the left side

CBCT was performed by a Gallileos set (Sirona Dental Systems GmbH, Bensheim, Hessen, Germany) and analyzed using Sidexis-XG software. We found a large unicystic radiolucency extending from the periapical area of 13 to the periapex of 16 (Figure 1). There was a complete absence of the maxillary sinus on the left side (Figure 1). 

**Figure 2:**
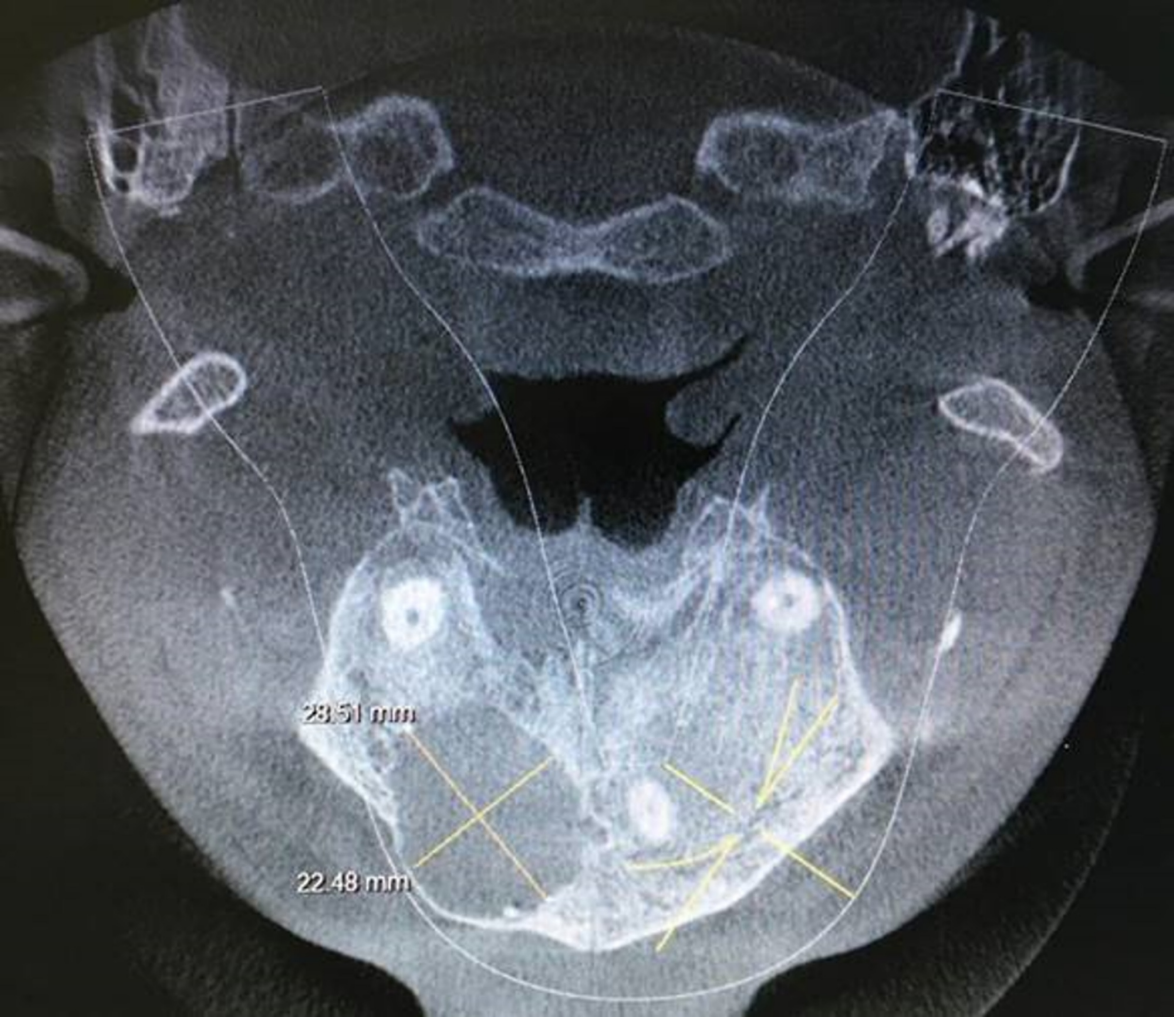
Axial section showing a cystic lesion 28.48 mm x 28.51 mm

An axial section showed a cystic lesion 28.48 mm x 28.51 mm (Figure 2).

**Figure 3:**
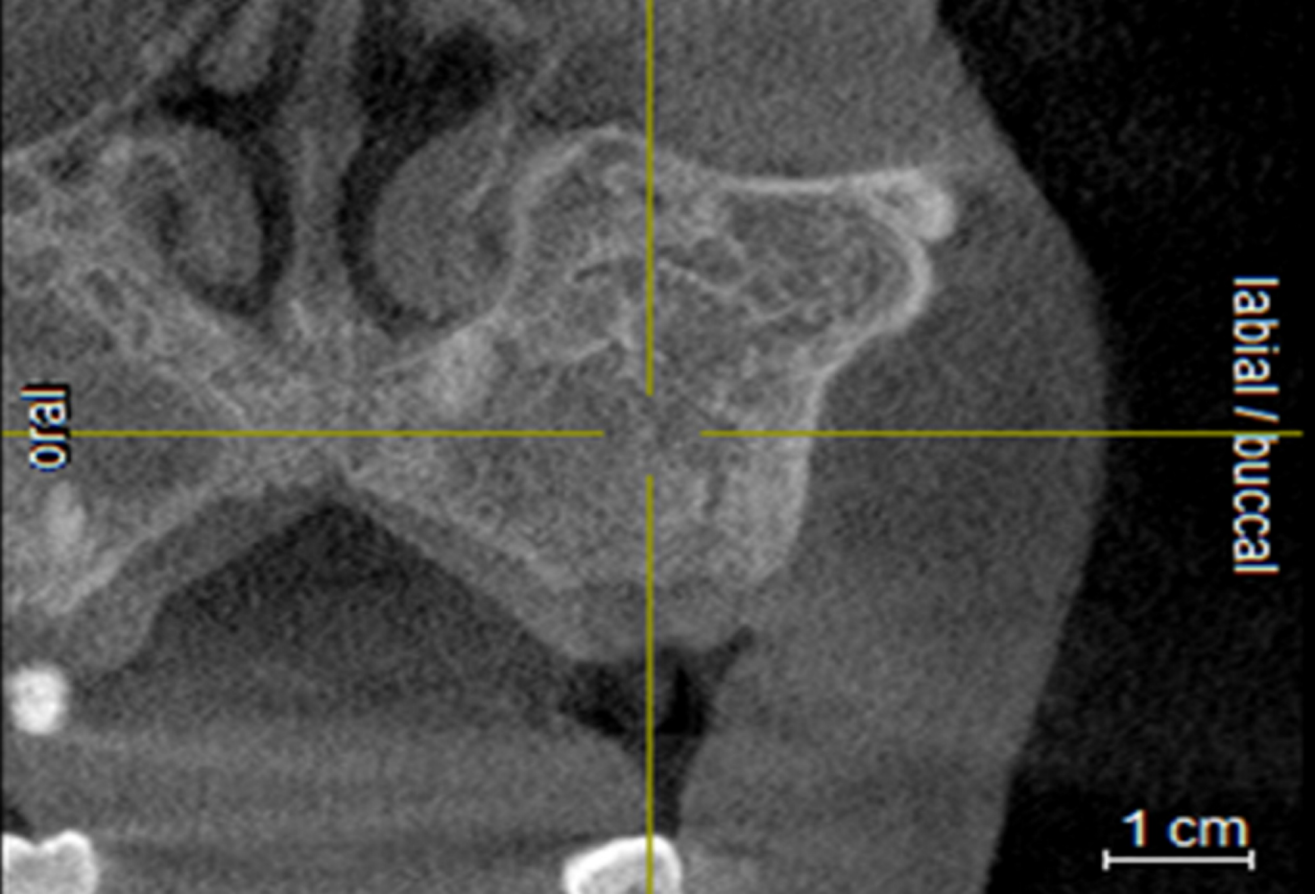
Sagittal slice showing a lesion with a superior extension

A sagittal slice showed a lesion with a superior extension (Figure 3). 

**Figure 4:**
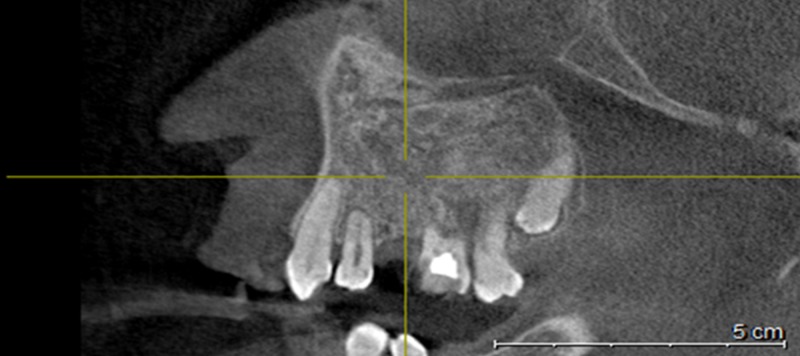
Coronal slice showing the complete absence of the maxillary sinus on the left side

 A complete absence of the maxillary sinus in the coronal slice is shown in Figure 4. 

**Figure 5:**
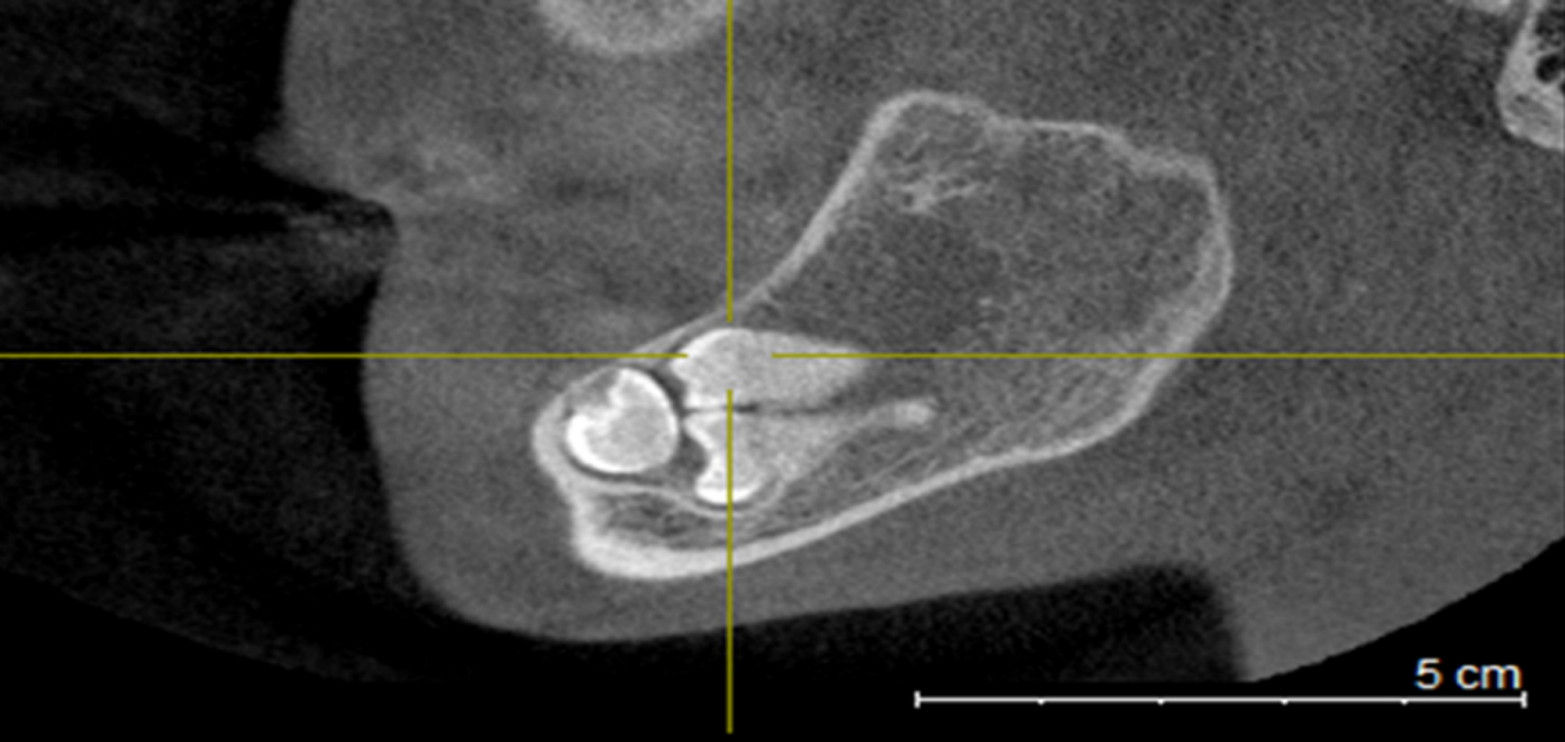
Sagittal slice showing 36, 37, and 38 on the left mandibular area with occlusal level of 36 below ‎the cervical line, and horizontally placed 37 and 38 in a sleeping position‎

Upon detailed evaluation of the CBCT results, we also found impacted maxillary supernumerary teeth palatal to 21 and 22, impacted 36, 37, and 38 on the left mandibular area with the occlusal level of 36 below the cervical line, and horizontally placed 37 and 38 in a sleeping position. In the mandibular right region, 47 and 48 were impacted (Figure 5). 

We performed a vitality test and found that 13 were non-vital. Fine needle aspiration cytology (FNAC) confirmed this as an inflammatory periapical cyst.

Radiologic diagnosis favored inflammatory periapical cyst in relation to 13, 14, 15, and 16. Type III aplasia of the left maxillary sinus was determined, with impacted supernumerary teeth in relation to 21 and 22, as well as impacted 36, 37, 38, 47, and 48.

The patient was referred to a higher center for further investigation and management.

## Discussion

MSH is less probable than sphenoid and frontal sinuses that can be congenital or acquired [1]. Some of the factors for aplasia or congenital hypoplasia include developmental arrest due to irradiation, infection and injury, first arch congenital syndrome, and developmental anomalies such as osteodysplasia, down syndrome, and craniosynostosis [8]. Interestingly, none of those factors were present in our case. Some factors are also liable for an acquired classification of MSH, such as trauma with deformity owing to fracture or surgery in the region of the sinus, cretinism, thalassemia, Wegener’s granuloma (inflammatory osteitis), and neoplasms that cause osteitis [1]. Our patient’s bloodwork results were normal and did not fit with the above classic factors.

Maxillary sinus aplasia or hypoplasia can trigger symptoms such as nasal discharge, headaches, facial pain, and speech problems. Most patients are asymptomatic and unaware of their circumstances [6]. Our patient reported experiencing an occasional headache and resonance in his voice, which indicated something about his condition was amiss. However, only the CBCT could confirm the diagnosis in this case. His right-side lesion was mimicking the maxillary sinus, and CBCT provided the confirmation that it was an apical cyst with the root tip of 13 and 14 inside the lesion. The radiographic differential diagnoses were fibrous dysplasia and eosinophilic granuloma. FNAC indicated that the mass was an inflammatory cystic lesion, which allowed us to arrive at the diagnosis of apical cyst of 13-16 and aplasia of the maxillary sinus on the left side.

## Conclusions

It is rare to find hypoplasia of the maxillary sinus. Often it will be found via routine CT imaging because it is asymptomatic. It is also rare to find hypoplasia involving two sinuses. An opacified maxillary antrum on plain films should be the differential diagnosis of maxillary sinus aplasia. Re-examination with CBCT in different slices may help confirm the aplasia involving the maxillary sinus. Routine CBCT evaluation of the maxillary sinus may play a major role in investigating maxillary sinus abnormalities, and further research into the feasibility and utility of this approach is warranted.
